# Hollow Polyaniline Microsphere/Fe_3_O_4_ Nanocomposite as an Effective Adsorbent for Removal of Arsenic from Water

**DOI:** 10.1038/s41598-020-61763-z

**Published:** 2020-03-18

**Authors:** Soumi Dutta, Kunal Manna, Suneel Kumar Srivastava, Ashok Kumar Gupta, Manoj Kumar Yadav

**Affiliations:** 1 School of Water Resources, Indian Institute of Technology Kharagpur, Kharagpur, 721302 India; 2 Department of Chemistry, Indian Institute of Technology Kharagpur, Kharagpur, 721302 India; 3 School of Energy Science and Engineering, Indian Institute of Technology Kharagpur, Kharagpur, 721302 India; 4 School of Environmental Science and Engineering, Indian Institute of Technology Kharagpur, Kharagpur, 721302 India

**Keywords:** Nanocomposites, Environmental sciences, Natural hazards

## Abstract

Polyaniline hollow microsphere (PNHM)/Fe_3_O_4_ magnetic nanocomposites have been synthesized by a novel strategy and characterized. Subsequently, PNHM/Fe_3_O_4_-40 (Fe_3_O_4_ content: 40 wt.%) was used as an adsorbent for the removal of arsenic (As) from the contaminated water. Our investigations showed 98–99% removal of As(III) and As(V) in the presence of PNHM/Fe_3_O_4_-40 following pseudo-second-order kinetics (*R*^2^ > 0.97) and equilibrium isotherm data fitting well with Freundlich isotherm (*R*^2^ > 0.98). The maximum adsorption capacity of As(III) and As(V) correspond to 28.27 and 83.08 mg g^−1^, respectively. A probable adsorption mechanism based on X-ray photoelectron spectroscopy analysis was also proposed involving monodentate-mononuclear/bidentate-binuclear As-Fe complex formation via legend exchange. In contrast to NO_3_^−^ and SO_4_^2−^ ions, the presence of PO_4_^3−^ and CO_3_^2−^ co-ions in contaminated water showed decrease in the adsorption capacity of As(III) due to the competitive adsorption. The regeneration and reusability studies of spent PNHM/Fe_3_O_4_-40 adsorbent showed ~83% of As(III) removal in the third adsorption cycle. PNHM/Fe_3_O_4_-40 was also found to be very effective in the removal of arsenic (<10 μg L^−1^) from naturally arsenic-contaminated groundwater sample.

## Introduction

Arsenic (As) remains one of the major sources of toxic pollutant in groundwater, affecting millions of people throughout the world. It associated into groundwater from several sources of natural and anthropogenic origins. The chronic exposure of arsenic contaminants in water beyond the World Health Organization (WHO) permissible limit (10 µg L^−1^) results in a serious toxicological and carcinogenic effect on human health^1^. It is also widely established that the presence of arsenite [As(III)] in water is more toxic and soluble compared to arsenate [As(V)]^2,3^. As a result, several technologies namely, co-precipitation, coagulation, oxidation, ion exchange, adsorption, membrane separation, etc. have been adopted for the treatment of such contaminated water^4,5^. However, many conventional and other approaches are not cost-effective and environmental friendly towards arsenic removal selectivity. For example, the precipitation of iron coagulation is cost-effective; however, it generates huge amounts of sludge, leading to secondary pollution problems^6^. Similarly, membrane separation exhibits high efficiency but involves high operational cost^6^. In this regard, the removal of toxic pollutants from water through adsorption has been receiving considerable attention due to its sludge-free operation, cost-effectiveness, high efficiency/selectivity, ease of use, and reusability facilities^7^.

Several nanoparticles, such as activated carbon, carbon nanotubes, graphene, manganese oxide, zinc oxide, titanium oxide, and ferric oxides emerged as effective nanoadsorbents give better performance compared to other conventional adsorbents in removal of arsenic, phosphate, selenium and nitrite anions, and other heavy metals from drinking water ^8–13^. In this context, high effective surface area, large number of active sites, high reactivity could contribute in efficient removal of pollutants from contaminant water^14^. Such nanoadsorbents need to be carefully synthesized in order to achieve maximum removal efficiency and easy separation. However, unavailability at economically affordable prices and toxicity, including environmental consequences, remain major concerns of nanomaterials^14^. Further, superior adsorption capacity and ease of separation/recovery of the nanoadsorbent are other prime requirements for an effective nanoadsorbent. The available literature suggested special affinity of iron-based adsorbents towards the arsenic removal due to their high selectivity to arsenic compounds from aqueous solutions^15,16^. Though the variety of iron oxides, such as Fe_3_O_4_^2^, α-Fe_2_O_3_^17^, γ-Fe_2_O_3_^18^, α-FeOOH^19^, β-FeOOH^20^, zero-valent iron^21^ have been employed in the removal of arsenic, only magnetic properties in some of these adsorbents are helpful for their separation by placing it under external magnetic field^18,22,23^. However, the higher tendency of aggregation of magnetic nanoparticles greatly diminishes availability, mobility, and transport to the contaminated site for *in situ* remediation^24^. Such agglomeration of bare magnetic particles in fixed-bed columns or any other dynamic flow system could result in pressure drops in on-field scale applications^25^. Therefore, the inclusion of some supporting materials in these magnetic adsorbents could be a better option for the removal of arsenic compounds from contaminated water.

Polyaniline (PANI) remains one of the most studied conducting polymer in recent times in water purifications^25^. Accordingly, PANI hybrids such as PANI/Polystyrene^26^, PANI/Rice husk^27^ have successfully been used in the separation of arsenic from water. The choice of PANI in these works is mainly guided by its lightweight, easy processability, flexibility, and excellent environmental stability^28^. However, separation of PANI powder absorbents is tedious and costly, owing to its intrinsic hydrophilicity^29^. Therefore, it is desirable to develop a polyaniline based magnetic adsorbents for the effective removal of arsenic and its easy separation after use^25,29,30^. Recently, the hollow morphology of PANI has been receiving considerable attention for multifaceted applications^28,31–33^. These are mainly ascribed to its porous structure, low effective density, lightweight, strong filling ability, chemical interest, thermal resistance, and reduced mass density^34^. It is anticipated that larger specific surface area and huge availability of inner space within the hollow PANI microsphere perhaps more beneficial in formation of corresponding nanocomposites with inorganic materials. Additionally, presence of its N containing amine and imine functional groups in PANI^33^ and magnetic Fe_3_O_4_ in hollow PANI microsphere/Fe_3_O_4_ (Referred as PNHM/Fe_3_O_4_) nano-adsorbent could play a dual role in the purification of water and separation/recovery, respectively. However, very limited work has existed on the fabrication of adsorbents derived from the combination of hollow PANI microsphere and inorganic counterparts for their applications in water treatment.

**Figure 1:**
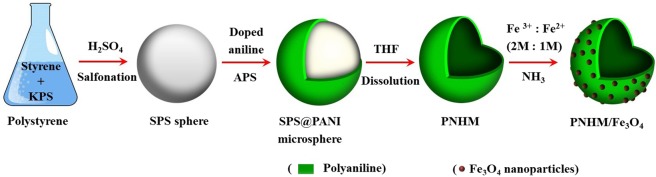
Schematic presentation of the synthesis process of PNHM/Fe_3_O_4_ composites (KPS: potassium peroxydisulfate, SPS: Sulfonated polystyrene, APS: Ammonium persulfate, THF: Tetrahydrofuran).

In view of this, present work is focused on fabrication of PANI hollow microsphere/Fe_3_O_4_ nano-adsorbent (Fig. 1). This is followed by its characterization by Field emissions scanning electron microscopy (FE-SEM), Transmission electron microscopy (TEM), Fourier transform infrared spectroscopy (FTIR), X-ray diffraction (XRD), X-ray photoelectron spectroscopy (XPS), Brunauer-Emmett-Teller (BET), and magnetic properties analysis to understand the morphology and composition of nanocomposites. Subsequently, PNHM/Fe_3_O_4_ has been used as an adsorbent in the removal of As(III) and As(V) from water and its application in real arsenic-contaminated groundwater sample. Effect of pH, initial concentration, contact time, dose, co-existing ions, regeneration, and recycle performances on the extent of arsenic removal from water has been studied. Finally, arsenic adsorption kinetics, isotherms, and probable adsorption mechanisms have also been investigated based on the experimental data.

## Results and Discussion

### Characterization of PNHM/Fe_3_O_4_ composites

#### FE-SEM

**Figure 2:**
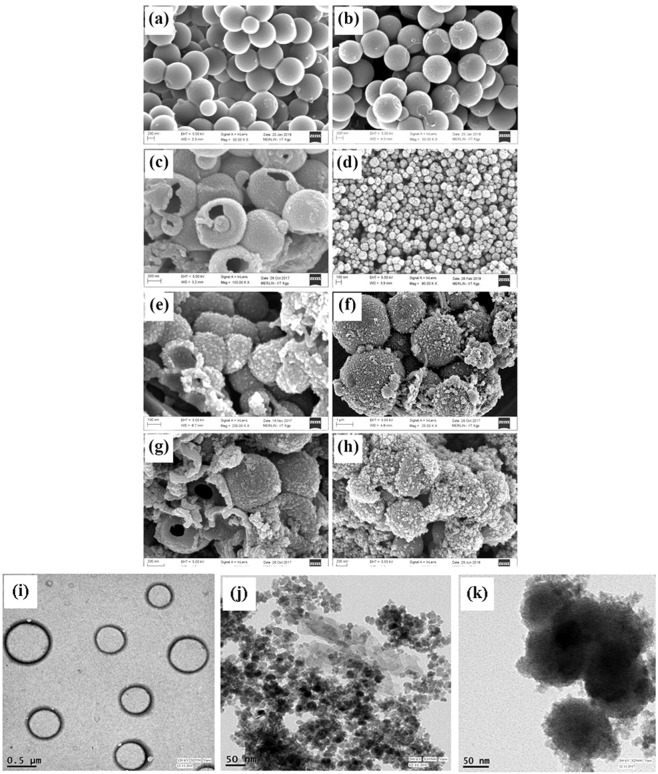
FE-SEM image of (**a**) PS spheres, (**b**) SPS@PANI, (**c**) PNHM, (**d**) Fe_3_O_4_ nanoparticles, (**e**) PNHM/Fe_3_O_4_-10, (**f**) PNHM/Fe_3_O_4_-20, (**g**) PNHM/Fe_3_O_4_-30, (**h**) PNHM/Fe_3_O_4_-40 composites. TEM images of (**i**) PNHM, (**j**) Fe_3_O_4_, (**k**) PNHM/Fe_3_O_4_-40 composites.

Figure 2(a–h) shows FE-SEM images of PS (Polystyrene) sphere, SPS@PANI (Sulfonated polystyrene@polyaniline), PNHM (polyaniline hollow microsphere), Fe_3_O_4_ and PNHM/Fe_3_O_4_ composites, respectively. The image of SPS@PANI clearly shows the coating of polyaniline on the surface of SPS spheres fabricated from PS microsphere of almost uniform diameters^32^. It is also evident from Fig. 2b that the smooth surface of PS sphere becomes rough on coating it with polyaniline. FE-SEM image in Fig. 2c depicts the formation of hollow spherical morphology of PNHM obtained by dissolving polystyrene core of SPS@PANI in THF (solvent). FE-SEM image in Fig. 2(d–h) shows more or less uniform dispersion of the Fe_3_O_4_ nanoparticles on the surface of PNHM.

#### TEM analysis

Morphology of PNHM, Fe_3_O_4_, and PNHM/Fe_3_O_4_-40 composites was further studied by TEM analysis, and corresponding findings are displayed in Fig. 2(i–k). The formation of hollow polyaniline microsphere is clearly inevitable on the expulsion of core polystyrene in THF solvent. TEM images of Fe_3_O_4_ indicated the presence of uniform nanoparticles with their average sizes of ~10 nm. Figure 2k demonstrated the dispersion of Fe_3_O_4_ particles on the surface of PNHM. It is anticipated that Fe_3_O_4_ nanoparticles are held on the surface of PNHM due to the interaction of hydroxyl groups on the surface of Fe_3_O_4_ and amino group of polyaniline^33^.

#### FTIR analysis

FTIR spectra of SPS, PNHM, and PNHM/Fe_3_O_4_ nanocomposites are displayed in Fig. S1 in Supplementary Information (SI). The peaks in PNHM correspond to C=C stretching vibration of the quinoid (1575 cm^−1^) and benzenoid rings (1466 cm^−1^)^35^. In addition, FTIR spectra also showed the appearance of the bands due to the C–N (1294 cm^−1^), C=N (1245 cm^−1^), and N=Q=N (1110 cm^−1^) vibration mode in doped polyaniline chain. These bands also appeared in all the PNHM/Fe_3_O_4_ nanocomposites, confirming the presence of polyaniline. Interestingly, spectra of PNHM/Fe_3_O_4_ showed disappearance of the signature peak of polystyrene corresponding to aryl C–H vibration bands (3081/3024 cm^−1^), alkyl C–H vibration bands (2919/2847 cm^−1^), benzene ring backbone vibration mode (1600/1497 cm^−1^) and out of plain C–H vibration (755/539 cm^−1^). This clearly signify complete expulsion of SPS from SPS@PANI due to its dissolution in THF. Additionally, the peak at 560 cm^−1^ corresponds to the Fe–O stretching vibration of Fe_3_O_4_^32^. All these findings further corroborated our findings based on FE-SEM and TEM on the successful decoration of Fe_3_O_4_ nanoparticles on the surface of PNHM.

#### X-ray diffraction

X-ray diffraction patters of PNHM, Fe_3_O_4_, PNHM/Fe_3_O_4_-10, PNHM/Fe_3_O_4_-20, PNHM/Fe_3_O_4_-30, and PNHM/Fe_3_O_4_-40 nanocomposites are displayed in Fig. S2. XRD of PNHM showed the presence of a broad peak centered around 2θ~25° due to periodically aligned chains in amorphous polymers^31^. The diffraction peaks in PNHM/Fe_3_O_4_ composites appeared at 2θ~30.23° (220), 35.58° (311), 43.24° (400), 53.68^0^ (422), 57.32^0^ (511) and 62.88^0^ (440) corresponding *fcc* spinel phase of Fe_3_O_4_ in accordance with JCPDS-19-0629^28^. Further, it is noted that the intensity of these peaks is considerably reduced with decreasing Fe_3_O_4_ content in PNHM.

#### BET analysis

BET surface area of PNHM/Fe_3_O_4_-40 was being calculated using the N_2_ adsorption-desorption method in the presence of liquid nitrogen, and corresponding plots are displayed in Fig. S3a and found to be ~64 m² g^−1^. It is noticed that the BET surface area of hollow morphological PNHM/Fe_3_O_4_-40 composite is higher than the reported in conventional PANI/Fe_3_O_4_ composite^12,36^. The Barrett–Joyner–Halenda (BJH) pore size distribution plot of PNHM/Fe_3_O_4_-40 is displayed in Fig. S3b. The average pore diameter and cumulative pore volume of PNHM/Fe_3_O_4_-40 were found to be in the range of 98.97–105.61 Å and 0.16–0.17 cm^3^ g^−1^, respectively.

#### Magnetic property analysis

**Figure 3:**
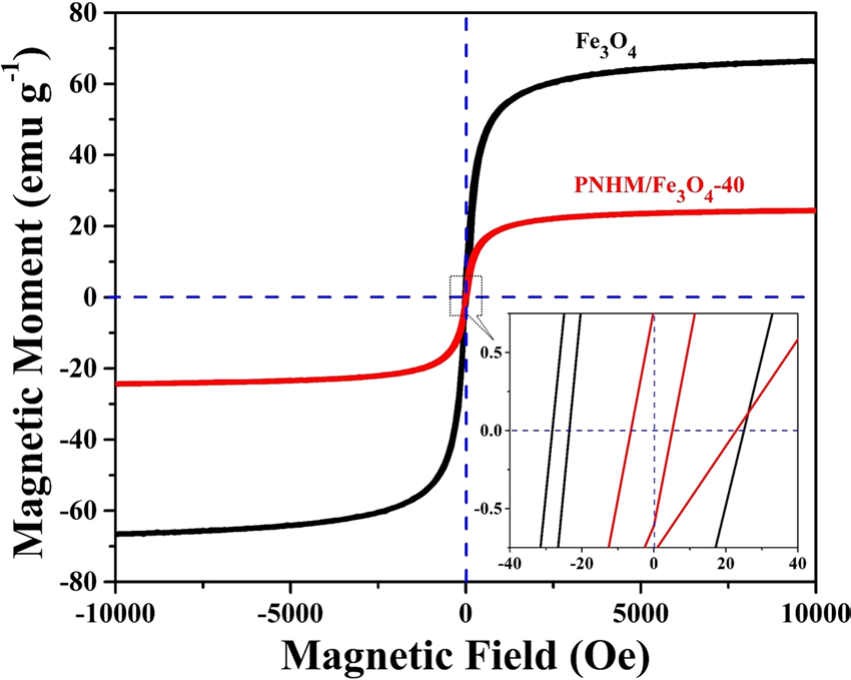
Magnetization curve of Fe_3_O_4_ and PNHM/Fe_3_O_4_-40 nanocomposites measured at room temperature.

Room temperature magnetization curves of Fe_3_O_4_ and PNHM/Fe_3_O_4_-40 in the presence of applied magnetic field ranging from −10000 Oe to +10000 Oe are displayed in Fig. 3 and corresponding saturation magnetization (M_s_), coercivity (H_c_), and remanence (M_r_) data are presented in Table S1 (Supplementary Information). The presence of hysteresis loops in Fe_3_O_4_ and PNHM/Fe_3_O_4_-40 confirmed ferromagnetic behaviors^34^. The saturation M_s_ value of PNHM/Fe_3_O_4_-40 (~24.39 emu g^−1^) was found to be somewhat smaller compared to Fe_3_O_4_ (~66.73 emu g^−1^). This is in all probability due to the contribution of non-magnetic polyaniline core wrapped by *in situ* grown Fe_3_O_4_ nanoparticles^37^. It is also noted that H_c_ values in PNHM/Fe_3_O_4_-40 (~30.84 Oe) and Fe_3_O_4_ (~31.34 Oe) remained almost unaltered. In contrast, M_r_ value of PNHM/Fe_3_O_4_-40 (~0.56 emu g^−1^) was considerably reduced in comparison to Fe_3_O_4_ (~4.29 emu g^−1^).

Figure S4a,b shows digital images of PNHM/Fe_3_O_4_-40 dispersed in aqueous solutions in the absence and presence of the external magnetic field. It is observed that after arsenic adsorption separation of PNHM/Fe_3_O_4_-40 from the solution can be achieved by applying the external magnetic field. Such magnetic recovery of adsorbent imparts an added advantage for reusing the spent material in the removal of arsenic from water.

### Application of PNHM/Fe_3_O_4_ as an adsorbent in the removal of As(III) and As(V) from contaminated water

#### Effect of Fe_3_O_4_ loading

The removal efficiency of As(III) and As(V) at different weight % loading of Fe_3_O_4_ in PNHM has been studied at a fixed dose (1 g L^−1^), and initial concentration (1000 μg L^−1^) and corresponding findings are displayed in Fig. S5. It is seen that As(III) and As(V) uptake increased significantly with increasing Fe_3_O_4_ content from 10 to 40 weight % in the PNHM/Fe_3_O_4_. However, the adsorption capacity of PNHM/Fe_3_O_4_ remains more or less unaltered at further higher loadings of Fe_3_O_4_. These is probably due to the accumulation and the coverage of excess Fe_3_O_4_ nanoparticles on the surface of hollow polyaniline sphere. In view of this, PNHM/Fe_3_O_4_ consisting of 40 weight % of Fe_3_O_4_ (optimum loading), has been employed for removal of As(III) and As(V) in all our investigations as discussed below.

#### Effect of the initial concentration of adsorbate

Figure S6 shows the effect of initial As(III) and As(V) concentration (100 to 20,000 μg L^−1^) on % removal of arsenic at 1 g L^−1^ dose of PNHM/Fe_3_O_4_-40. It is noticed that the efficiency of arsenic removal is higher in lower concentration of arsenic and vice versa. The observed successive decrease in the % removal of As(III) and As(V) with adsorbate loadings could result in the saturation of binding capacity of the adsorbent. Alternatively, this could be attributed to the saturation of active sites of the adsorbent at higher concentration^38^.

#### Effect of adsorbent dose

**Figure 4:**
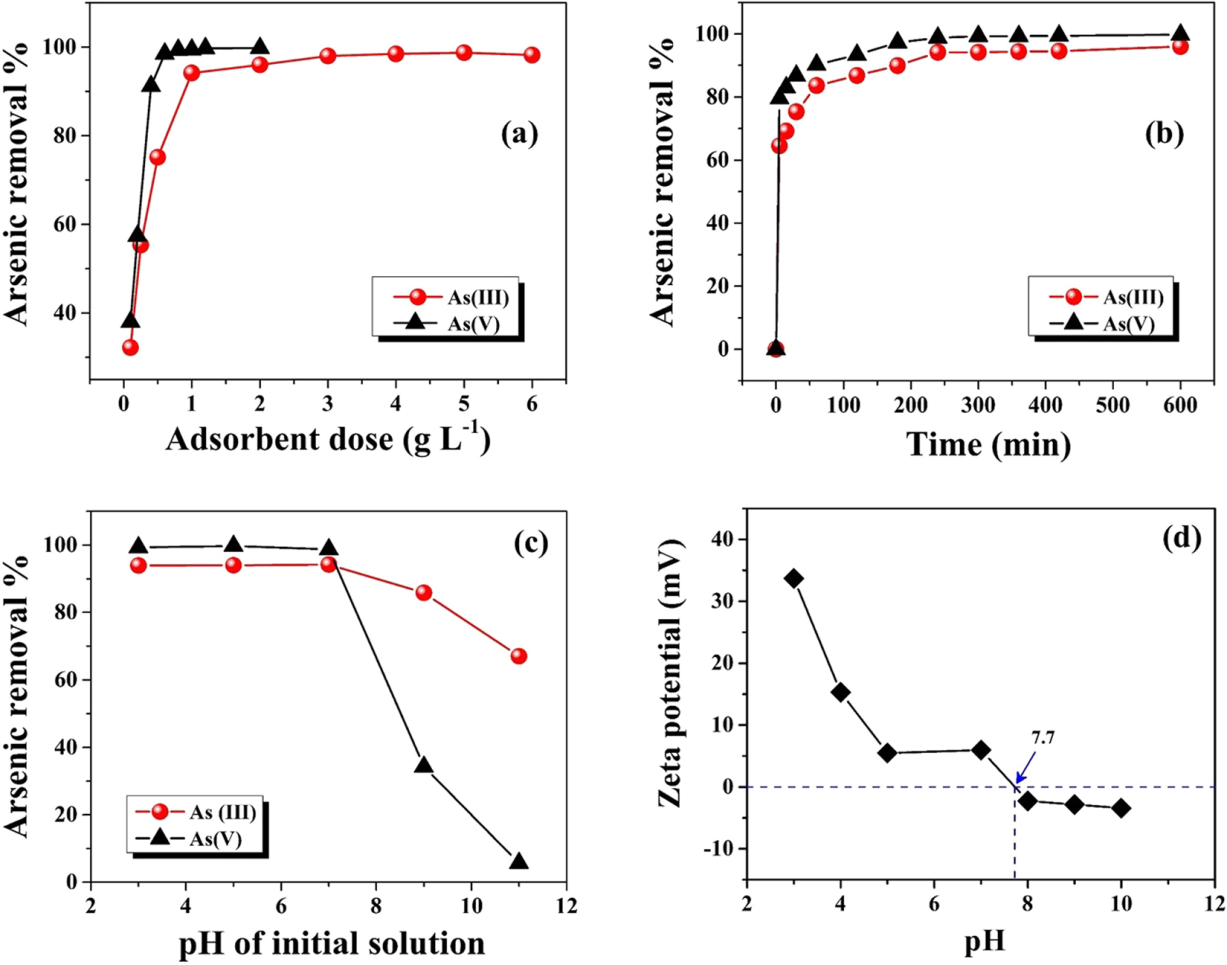
(**a**) Effect of adsorbent dose (Experimental conditions: C_0_: 1000 µg L^−1^; pH~7; contact time: 240 min; T: 300 ± 3 K), (**b**) Effect of contact time (Experimental conditions: adsorbent dose: 1 g L^−1^; C_0_: 1000 µg L^−1^; pH~7; T: 300 ± 3 K), (**c**) Influence of initial solutions pH (Experimental conditions: adsorbent dose: 1 g L^−1^; C_0_: 1000 µg L^−1^; contact time: 240 min; T: 300 ± 3 K) on As(III) and As(V) removal efficiency using PNHM/Fe_3_O_4_-40. (**d**) ζ–potential of PNMH/Fe_3_O_4_-40 under various pH conditions.

The effect of adsorbent dose on % removal of As(III) and As(V) is displayed in Fig. 4a. It is noted that arsenic removal efficiency initially increases with the increase of adsorbent dose due to the availability of more adsorption sites on the surface of PNHM/Fe_3_O_4_-40^25^. Figure 4a also indicated ~32 to 98% removal of As(III) corresponding to the adsorbent dose of 0.1 to 6 g L^−1^, respectively. Similar studies on As(V) removal showed an increase in its removal from ~38 to 99% (dose: 0.1–2 g L^−1^). These findings also indicated ~94 and 99% removal of As(III) and As(V) with adsorbent dose of 1 g L^−1^ (optimum dose) at neutral pH, respectively. Subsequently, % removal of arsenic remained more or less unchanged at higher adsorbent doses (1 g L^−1^ onwards). These could be ascribed to the binding of almost all arsenic species on the PNHM/Fe_3_O_4_-40 surface and establishment of equilibrium between the arsenic species on the adsorbent surface and arsenic solution^39^.

#### Effect of contact time

Figure 4b represents the variation in % removal of As(III) and As(V) with the change of contact time at a constant adsorbent dose (1 g L^−1^) and initial arsenic concentration (1000 µg L^−1^). It is noted that in both cases arsenic removal increased very rapidly up to 30 min (As(III): 76%, As(V): 87%), this could be ascribed to the availability of high concentration gradient and presence of more active sites on the surface of PNHM/Fe_3_O_4_-40^40,41^. Subsequently, % removal of arsenic getting slower with the increase of contact time and finally attained equilibrium (As(III): 94%, As(V): 99%) at about 240 min. Further, increase of time (beyond 240 min) % removal of arsenic was not increased considerably, which stipulated that limited mass transfer of the adsorbate molecules from the bulk arsenic solution to the external surface of adsorbent (PNHM/Fe_3_O_4_-40)^41^. In addition, time study experiments have also been conducted at higher adsorbent dose, keeping fixed adsorbate concentration, and corresponding findings are displayed in Fig S7. It is noted that % removal and the corresponding rate of arsenic adsorption on the surface of PNHM/Fe_3_O_4_-40 increases with increasing the adsorbent dose from 1 to 5 g L^−1^. These could be ascribed to the fact that with increase in the adsorbent dose, there is an increase in number of active sites, which enhances the adsorption of arsenic^25^.

#### Effect of pH

Effect of pH on removal of As(III) and As(V) has been studied by changing the initial solution pH in the range of 3–11 at a fixed initial arsenic concentration (1000 µg L^–1^) and dose (1 g L^−1^) and corresponding outcomes are presented in Fig. 4c. These findings showed the removal of ~94% As(III) and 98% As(V) in the optimum pH range of 3–7, followed by a continuous decrease at higher pH ranges (8–11). Most likely, adsorption of arsenic species are controlled by the surface charge of the adsorbent, which is largely dependent on the pH of the solution^21,42,43^. In order to strengthen this contention, point of zero charges (PZC) of the surface in material (PNHM/Fe_3_O_4_-40) was evaluated by studying the variation of ζ–potential versus pH and corresponding findings in Fig. 4d show PZC of PNHM/Fe_3_O_4_-40 at pH = 7.7 (pH_PZC_). The variation of pH < pH_PZC_ suggested adsorbent surface to be positively charged. Further, the presence of As(III) vis-à-vis pH of the solution can be explained in terms of the arsenite species and equilibrium constant as below^38,44^:

1 $${{\rm{H}}}_{3}{{\rm{AsO}}}_{3}\leftrightarrow {{\rm{H}}}_{2}{{{\rm{AsO}}}_{3}}^{-}+{{\rm{H}}}^{+};{\rm{p}}{{K}}_{{a}}=9.23$$

2 $${{\rm{H}}}_{2}{{{\rm{AsO}}}_{3}}^{\mbox{--}}\leftrightarrow {{{\rm{HAsO}}}_{3}}^{2-}+{{\rm{H}}}^{+};{\rm{p}}{K}_{a}=12.1$$

3 $${{{\rm{HAsO}}}_{3}}^{2\mbox{--}}\leftrightarrow {{{\rm{AsO}}}_{3}}^{3-}+{{\rm{H}}}^{+};{\rm{p}}{K}_{a}=12.7$$

In addition, possible arsenate species present under different pH conditions along with their equilibrium constant can also be described below^44^:

4 $${{\rm{H}}}_{3}{{\rm{AsO}}}_{4}\leftrightarrow {{\rm{H}}}_{2}{{{\rm{AsO}}}_{4}}^{-}+{{\rm{H}}}^{+};{\rm{p}}{K}_{a}=2.22$$

5 $${{\rm{H}}}_{2}{{{\rm{AsO}}}_{4}}^{\mbox{--}}\leftrightarrow {{{\rm{HAsO}}}_{4}}^{2-}+{{\rm{H}}}^{+};{\rm{p}}{K}_{a}=6.98$$

6 $${{{\rm{HAsO}}}_{4}}^{2\mbox{--}}\leftrightarrow {{{\rm{AsO}}}_{4}}^{3-}+{{\rm{H}}}^{+};{\rm{p}}{K}_{a}=11.53$$

Therefore, it is anticipated that at pH < 7.7, predominated arsenite and arsenate species are neutrally (H_3_AsO_3_) and negatively charged (H_2_AsO_4_^–^ and HAsO_4_^2−^), respectively. As a result, adsorption of As(V) is accompanied by electrostatic attraction between negatively charged As(V) species and positively charged surface of PNHM/Fe_3_O_4_-40. In contrast, there exist unfavorable electrostatic interactions between the non-ionic As(III) species and the positively charged adsorbent surface. Thus, good removal of As(III) in our case may be due to the oxidation reaction of As(III) to As(V) followed by sorption on the positive surface of PNHM/Fe_3_O_4_-40^45,46^. Alternatively, the possibility of adsorption of As(III) on the surface of PNHM/Fe_3_O_4_-40 via surface complexation rather than electrostatic interactions also cannot be ruled out^47^. At pH > 9, adsorption is inhibited due to the electrostatic repulsion between negatively charged arsenite/arsenate species and negatively charged surface of PNHM/Fe_3_O_4_-40 in the presence of excessive amount of OH^−^ ^25,48,49^.

#### Adsorption kinetics

**Figure 5:**
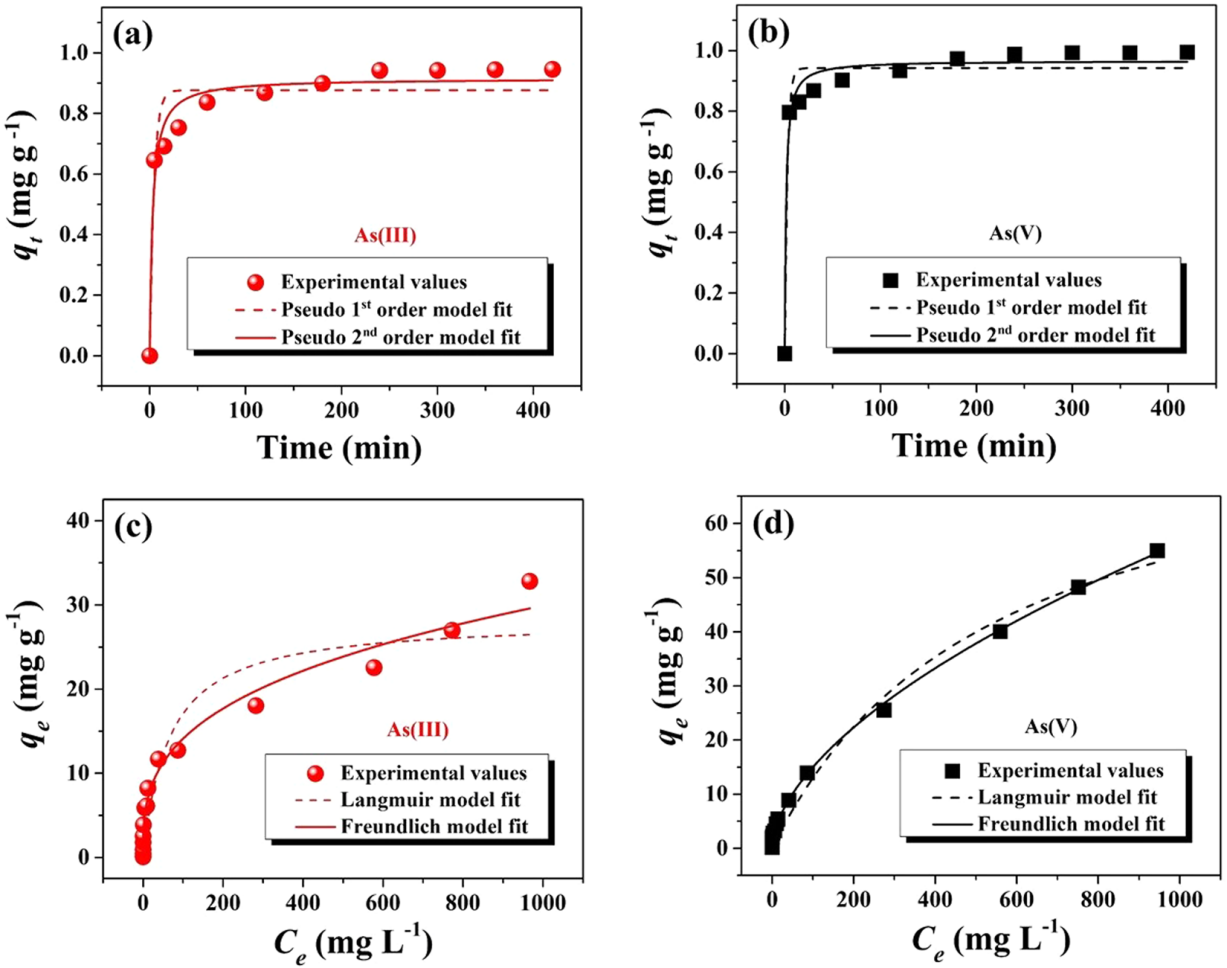
Fitting of kinetic data in pseudo-first-order and pseudo-second-order models for (**a**) As(III) and (**b**) As(V). Experimental values fitted in Langmuir and Freundlich isotherm model for (**c**) As(III) and (**d**) As(V) adsorption on PNHM/Fe_3_O_4_-40 at neutral pH.

The rate of arsenic adsorption on the surface of PNHM/Fe_3_O_4_-40 was studying, considering pseudo-first-order and pseudo-second-order kinetic models as described under Supplementary information-1 (SI–1). Figure 5a,b shows the fittings of pseudo-first-order and second-order kinetic model for As(III) and As(V) adsorption, respectively. These findings demonstrated the arsenic adsorption process considering the following order: 0–30 min (rapid), 30–180 min (gradual), and 240–360 min (equilibrium). Table S2 provides kinetic parameter data for As(III) and As(V) series, respectively. The higher correlation coefficient (*R*^2^) values (As(III): 0.97, As(V): 0.98) and smaller residual root mean square error (RMSE) value (As(III): 0.05, As(V): 0.04) clearly indicate adsorption kinetics for As(III) and As(V) followed the pseudo-second-order model. The information on rate-limiting steps could be described according to the Weber-Morris equation as follows^50^:

7 $${q}_{t}={k}_{d}{t}^{0.5}+C$$

where *t* is contact time (min), *k*_*d*_ and *C* indicates the intra-particle diffusion rate constant (mg g^−1^ min^−0.5^) and thickness of the boundary layer, respectively. If the plot of *q*_*t*_ vs. *t*^*0.5*^ is linear and passing through the origin, then sorption mechanisms should follow intraparticle diffusion as a rate-limiting step. Figure S8 shows variation of *q*_*t*_ versus *t*^0.5^ for sorption of As(III) and As(V) on the surface of PNHM/Fe_3_O_4_-40. Each plot shows multi-linearity, which consists of two distinct slopes, and neither of them passed through the origin. These suggest that the sorption process could be controlled by more than one mechanism^11,48^. Here, the steeper slope indicates the rapid extraction of solute, which is directed by surface or film diffusion. The gradual slope, where the equilibrium has been reached, indicates slow adsorption attributed to intra-particle or pore diffusion^50,51^. Corresponding intra-particle diffusion model parameters for As(III) and As(V) are presented in Table S2.

#### Adsorption isotherms

Adsorption equilibrium phenomenon between solid phase (PNHM/Fe_3_O_4_-40) and liquid phase (As(III) and As(V)) have been studied based on Langmuir and Freundlich isotherms model (SI-2), and corresponding isotherms are displayed in Fig. 5c,d, respectively. The related model parameters were calculated and recorded in Table S3. The higher *R*^2^ (As(III): 0.98, As(V): 0.99) and smaller RMSE values (As(III): 1.26, As(V): 0.73) suggested relatively better fitting of Freundlich isotherm compared to Langmuir. Further, Freundlich isotherm parameter *1/n* < 1 suggested the adsorption process to be favorable as well as the heterogeneity of the adsorbent sites^52,53^. These findings also indicated adsorption of As(III) and As(V) on PNHM/Fe_3_O_4_-40 surface no more limited to monolayer adsorption and could successfully be applied even to multilayer adsorption over the heterogeneous surface^54,55^. Further, *Yang*^56^ and *Boparai et al*.^50^, reported applicability of Freundlich isotherm equation to both monolayer adsorption (chemisorption) as well as multilayer adsorption (van der Waals adsorption). It is proposed that stronger binding sites on the heterogeneous surface of the adsorbent are occupied first until adsorption energy is exponentially decreased upon the completion of adsorption process^54,57^. Similar behavior is expected in our case because of the heterogeneity of PNHM/Fe_3_O_4_-40 surface due to the coating Fe_3_O_4_ nanoparticles. According to *Mandal et al*.^53^, adsorption of As(III) on zirconium polyacrylamide hybrid followed Freundlich model involving electrostatic attraction and surface complexation formation between positively charged surface hydroxyl group and arsenic species. Similarly, *Setyonoa et al*.^58^ observed stronger interaction of CeO_2_ on MC-2 with As(III) and As(V) through the formation of inner-sphere surface complex. *Tuna et al*.^59^ also suggested adsorption of As(V) through ligand exchange mechanism and formation of an inner-sphere surface complex. In another work, contribution of ion exchange/electrostatic attraction (physisorption) and surface complexation (chemisorption) was suggested to be operative at different extent in the adsorption of arsenic on magnetite modified fly ash^60^.

Freundlich and Langmuir’s isotherms provide the value of relative adsorption capacity (*K*_*f*_) and maximum adsorption capacity (*Q*_*m*_), respectively. In view of this, Table S4 provides a comparison of maximum adsorption capacity of arsenic on PNHM/Fe_3_O_4_-40 along with other reported adsorbents (Fe_3_O_4_@polyaniline, porous Fe_3_O_4_, commercial Fe_3_O_4_, Fe_2_O_3_@C, polyaniline/polystyrene nanocomposite, etc.). It is noted that *Q*_*m*_ values for As(III) adsorption on PNHM/Fe_3_O_4_-40 corresponds to 28.27 mg g^−1^ and comparable to Fe_2_O_3_@C (29.4 mg g^−1^)^61^, unlike other reported adsorbents. However, *Q*_*m*_ value for the adsorption of As(V) on PNHM/Fe_3_O_4_-40 (83.08 mg g^−1^) was found to be much higher compared to other adsorbents at neutral pH. Such increase in the adsorption capacity is in all probability due to the dispersion of Fe_3_O_4_ nanoparticles on the higher surface area of the hollow polyaniline microsphere.

#### Effect of co-existing ions

The various interfering anions including sulfate (SO_4_^2−^), carbonate (CO_3_^2−^), nitrate (NO_3_^−^), and phosphate (PO_4_^3−^) present in the groundwater could significantly influence the arsenic removal efficiency^20^. Accordingly, investigations have been made to comprehend the interferences of co-existing ions in the adsorption of arsenite on PNHM/Fe_3_O_4_-40. This experiment is conducted by varying the concentrations of SO_4_^2–^, CO_3_^2–^, NO_3_^–^ and PO_4_^3−^ between 10 to 100 mg L^–1^ at constant arsenite concentrations of 1000 µg L^–1^ and PNHM/Fe_3_O_4_-40 doses of 1 g L^−1^ at pH~7. The results are displayed in Fig. S9 and it shows no significant effects on arsenite uptake in the presence of SO_4_^2−^ and NO_3_^−^ by PNHM/Fe_3_O_4_-40. On the contrary, decrease in the arsenite removal efficiency is observed in the presence of PO_4_^3−^ and CO_3_^2−^ co-ions. It is anticipated that adsorption of PO_4_^3−^ and CO_3_^2−^ ions present along with arsenic species compete for the same sites in PNHM/Fe_3_O_4_-40. As a result, in presence of PO_4_^3−^ and CO_3_^2−^ ion (concentrations ranging from 0 to 100 mg L^−1^), arsenite removal efficiency considerably reduced from ~94 to 59% and ~94 to 63%, respectively. Further, it was noted that arsenite adsorption in the presence of co-existing ions follows the order NO_3_^−^ < SO_4_^2−^ < CO_3_^2−^ < PO_4_^3−^ and in agreement with that reported by many other researchers^20,21,25,44^. XPS analysis of used PNHM/Fe_3_O_4_-40 adsorbent already established the partial oxidation of arsenite to arsenate. In view of this, the high selectivity of PNHM/Fe_3_O_4_ toward arsenic species (arsenite/arsenate) is likely to originate from combined effects of hydrogen bonding and ligand exchange mechanisms^16,62^.

Arsenic and phosphoric acid are triprotic acids and exhibit almost similar structure and chemical properties^6,9^. Therefore, competitive adsorption of arsenate and phosphate is anticipated due to their comparable dissociation values (p*K*_b_) through complex formation between protonated −FeOH_2_^+^ functional group in PNHM/Fe_3_O_4_ and AsO_4_^3−^/PO_4_^3−^ (in water) via hydrogen bonding^4,9,16^. Further, decrease in the % of As(III) removal in the presence of high concentration of CO_3_^2−^ ion could be attributed to the inhibitory adsorption effect of As(III) compared to CO_3_^2−^ on the surface of PNHM/Fe_3_O_4_-40. Alternatively, the possibility of formation of arsenic–carbonate As(CO_3_)^2−^, As(CO_3_)(OH)^2−^, and AsCO_3_^+^ complex in the presence of high concentration of CO_3_^2−^ could also account for the observed decrease in arsenic adsorption^63^. In contrast, unaltered selectivity of arsenic in the presence of SO_4_^2−^ (p*K*_b_ = 7.04) could result in preferential adsorption of AsO_4_^3−^ (p*K*_b_ = 2.5) on the surface of PNHM/Fe_3_O_4_-40 through hydrogen bonding^64^. Similarly, the unchanged removal efficiency of arsenic in arsenic/NO_3_^−^ contaminated water could be ascribed to weaker competitive adsorption ability of NO_3_^−^ co-ion on PNHM/Fe_3_O_4_^62^.

#### Mechanism of arsenic adsorption

**Figure 6:**
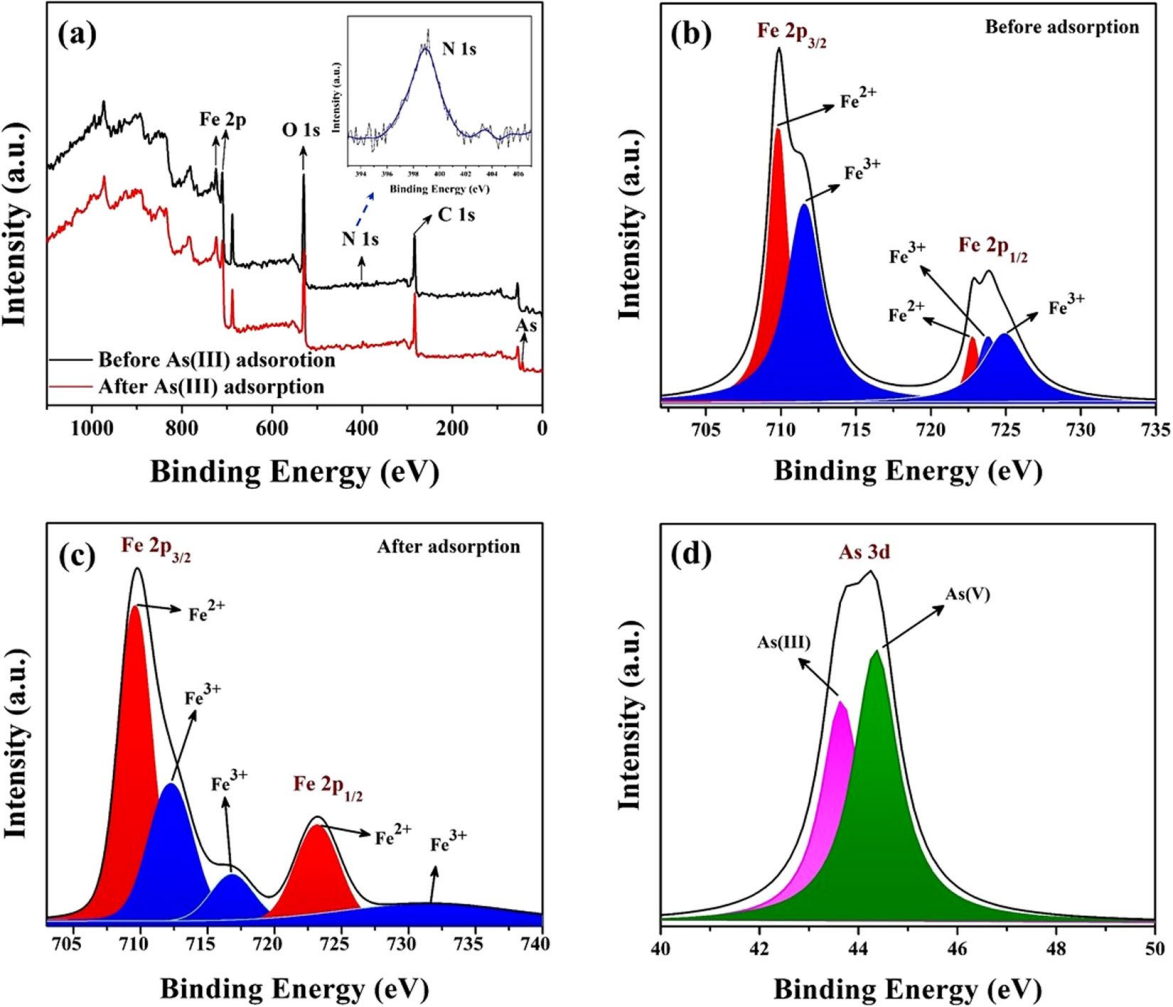
(**a**) XPS survey spectra of PNHM/Fe_3_O_4_-40 before and after As(III) adsorption, (**b**) Fe 2p high resolution core level XPS spectra of PNHM/Fe_3_O_4_-40 before As(III) adsorption, (**c**) Fe 2p high resolution core level XPS spectra of PNHM/Fe_3_O_4_-40 after As(III) adsorption, (**d**) As 3d XPS spectra of PNHM/Fe_3_O_4_-40 after As(III) adsorption.

The possible mechanism of arsenic adsorption on PNHM/Fe_3_O_4_-40 could be proposed based on FTIR and XPS study, as shown in Fig. S10 and Fig. 6, respectively. FTIR spectra show the shifting of Fe−O band (543 cm^−1^) in spent PNHM/Fe_3_O_4_-40 compared to its pure counterpart (584 cm^−1^). These could be ascribed to the interaction of wrapped Fe_3_O_4_ nanoparticles at the exterior surface of PNHM with As(III)^42^. However, the band corresponding to surface water molecules (~3400 cm^−1^) and hydrogen-bonded surface −OH groups remain more or less unaltered, which is an all probability due to their non-involvement for adsorption of As(III)^20^. Further, in-plane deformation vibration of −OH bond in Fe−OH (~1375 cm^−1^) almost disappeared in the spent adsorbent. These could be attributed to the substitution of −OH groups with adsorbed arsenic species^20^. Additionally, XPS analysis has also been widely utilized in understanding the mechanism of arsenic adsorption on adsorbent (PNHM/Fe_3_O_4_-40). The corresponding survey spectra of PNHM/Fe_3_O_4_-40, core level spectrum of Fe 2p, and As 3d, before and after adsorption study are displayed in Fig. 6. The appearance of peaks in survey spectra in Fig. 6a corresponds to O 1 s (~530 eV), N 1 s (~399 eV), C 1 s (~258 eV), and Fe 2p (~723 eV and ~710 eV) indicating the presences of PANI as well as Fe_3_O_4_ in the composites material^25,28^. In addition, another peak also appeared around ~42–46 eV in spent adsorbent, in all possibility due to the presence of As(III) on the surface of PNHM/Fe_3_O_4_-40^25,51^. According to available literature, spectra of Fe 2p consist of Fe^2+^ as well as Fe^3+^ ^51,65^. The spectra of pure PNHM/Fe_3_O_4_-40 in Fig. 6b shows the presence of peak 709.8 eV (Fe 2p_3/2_) and 722.8 eV (Fe 2p_1/2_) which attributed to Fe^2+^; the peaks at 711.6 eV (Fe 2p_3/2_) and 723.8 eV, 725 eV (Fe 2p_1/2_) were assigned to Fe^3+^ ^25,51,65^. After adsorption of As(III) on the surface of PNHM/Fe_3_O_4_-40, corresponding peaks are shifted in Fe^3+^ (~712.25, 717, and 730 eV) and Fe^2+^ (~709.5, 723.25 eV) as depicted in Fig. 6c. The calculation based on XPS shows the presence of relatively more Fe^2+^ (~58.55%) compared to Fe^3+^ content (~41.44%) in spent adsorbent due to redox reaction after adsorption of As(III)^51^. Further, Fig. 6d shows the appearance of two peaks in XPS spectra of As 3d due to the presence of both As(III) (~43.70 eV) and As(V) (~44.40 eV) and their content corresponding to ~42.02 and 57.98%, respectively^13,51^. These findings further strengthen the fact that Fe^3+^ present in PNHM/Fe_3_O_4_-40 plays an imperative role in oxidizing As(III) (more toxic) to As(V) (less toxic), followed by adsorption^51,66^.

**Figure 7:**
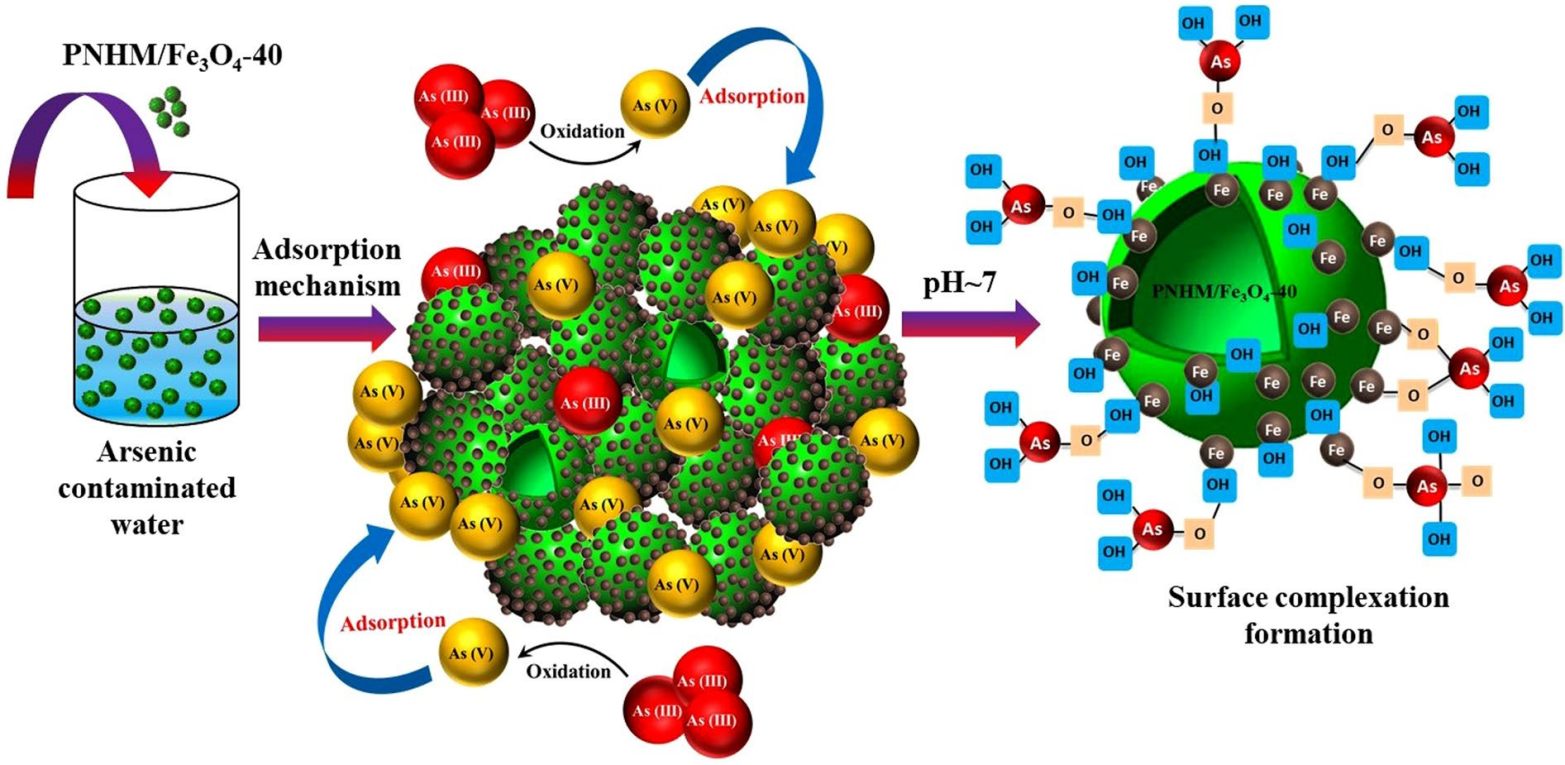
Schematic representation of arsenic adsorption mechanism in aqueous solution.

Considering all the above facts probable sorption mechanism can be proposed considering the formation of monodentate-mononuclear and bidentate-binuclear As–Fe complexes via ligand–exchange mechanism^42,67^ as illustrated in Fig. S11. Accordingly, arsenic oxyanion (AsO_3_^3−^) is adsorbed on the surface of adsorbent by forming a complex with protonated surface hydroxyl (−FeOH_2_^+^) groups originating from PNHM/Fe_3_O_4_-40 at pH < 7.7 (PZC), illustrated schematically in Fig. 7^42,51^. In all probability, the higher extent of inner-sphere complex formation via hydrogen bonding, as well as electrostatic attraction between arsenic species and surface hydroxyl group in PNHM/Fe_3_O_4_-40, could account for such superior adsorption capacity compared to other composite adsorbents^16,51,53^.

#### Desorption and reusability study

Desorption study was performed to confirm the reusability and applicability of the PNMH/Fe_3_O_4_-40 as adsorbent. This is executed by using different concentrations of strong alkaline (NaOH) solutions to remove the adsorbed arsenic from PNMH/Fe_3_O_4_-40 following the method as reported earlier^20,25,38^. Figure S12a shows the variations of As(III) desorption from PNHM/Fe_3_O_4_-40 by 0.1 and 0.5 M NaOH solutions. It is noted that the desorption of As(III) is achieved 75.76 and 82.32% using 0.1 M and 0.5 M NaOH, respectively.

The adsorption/desorption cycles were also performed to investigate the reusability of the as-synthesized PNMH/Fe_3_O_4_-40 for the removal of As(III). Figure S12b demonstrates the variation of As(III) removal percentages versus the number of adsorption cycles using regenerated PNMH/Fe_3_O_4_-40. It is observed that ~83% of As(III) removal was achieved in the third adsorption cycle at a dose of 1 g L^−1^ and initial As(III) concentration of 1000 μg L^−1^. It is noted that the removal efficiency of As(III) using regenerated PNMH/Fe_3_O_4_-40 is higher than many other reported adsorbents^38,42^. These findings also indicated that PNMH/Fe_3_O_4_-40 could be used many more times successfully after NaOH treatment in a sustainable manner.

Leaching of iron from PNHM/Fe_3_O_4_-40 in water is also tested, and it is observed that the presence of iron in treated water is below the WHO permissible limit (0.3 mg L^–1^). These findings suggested that PNMH/Fe_3_O_4_-40 could be safely used for aqueous arsenic remediation.

### Removal of arsenic from naturally arsenic-contaminated groundwater sample

**Figure 8:**
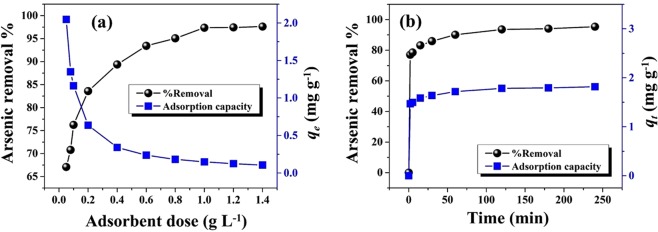
Effect of (**a**) PNHM/Fe_3_O_4_-40 dose, (**b**) Contact time on the removal of arsenic from naturally contaminated groundwater sample.

Arsenic contaminated groundwater sample collected from Nalkora area of Basirhat subdivision under North 24 Parganas districts (West Bengal, India) was subjected to arsenic removal in the presence of PNMH/Fe_3_O_4_-40 adsorbent. Table S5 records initial arsenic concentration and other physicochemical parameters of the arsenic-contaminated groundwater sample. Figure 8 shows the variation of % removal and adsorption capacity of arsenic with varying adsorbent doses (PNHM/Fe_3_O_4_-40). Results show that 0.8 g L^–1^ of dose is sufficient to bring down the arsenic concentration below the WHO specified drinking water standards. In addition, experiments were also made to study % removal and adsorption capacity of arsenic in real groundwater sample by varying contact time (2 to 240 min) at a dose of 0.8 g L^−1^ (Fig. 8). Our finding shows 78% arsenic removal achieved in 2 min and end to approach equilibrium in the range of 60 min (90%) to 240 min (96%). All these investigations successfully demonstrated the applicability of prepared adsorbents (PNHM/Fe_3_O_4_-40) for the removal of arsenic from real-life arsenic-contaminated groundwater samples.

## Conclusion

Fe_3_O_4_ coated polyaniline hollow microsphere composites were fabricated, characterized and used as adsorbent in removal of As(III) and As(V) from water. The batch study experiments under neutral pH and 1 g L^−1^ dose of 40 wt.% Fe_3_O_4_ loaded polyaniline (PNHM/Fe_3_O_4_-40) indicated the removal of ~94 and 99% of As(III) and As(V), respectively. The removal efficiency of As(III) was found to be effected in presence of CO_3_^2−^ and PO_4_^3−^ co-existing ions in contrast to NO_3_^−^ and SO_4_^2−^ ions. The adsorption of As(III) and As(V) on PNHM/Fe_3_O_4_-40 followed the pseudo-second-order kinetics and Freundlich isotherm. Moreover, the formation of monodentate-mononuclear/bidentate-binuclear As-Fe surface complex is accounted for the adsorption of arsenic species on the surface of PNHM/Fe_3_O_4_-40. The desorption study demonstrated successfully removal of arsenic in high basic environment from spent adsorbent and could be effectively reused several times. The PNHM/Fe_3_O_4_-40 composite was also found to be very effective in removal of arsenic from naturally contaminated groundwater samples. In view of the aforementioned outcomes, PNHM/Fe_3_O_4_-40 composites acted as a promising adsorbent in the removal of arsenic from contaminated water.

## Methods

### Materials

Analytical grade chemicals and reagents were used in this study. Styrene [C_8_H_8_], potassium peroxydisulfate (KPS) [K_2_S_2_O_8_], tetrahydrofuran (THF) [C_4_H_8_O], aniline monomer [C_6_H_5_NH_2_], ammonium persulfate (APS) [(NH_4_)_2_S_2_O_8_], ferric chloride hexahydrate [FeCl_3_.6H_2_O], ferrous sulphate heptahydrate [FeSO_4_.7H_2_O], hydrochloric acid [HCl], ammonia solution [NH_3_], sulfuric acid [H_2_SO_4_], methanol [CH_3_OH] and ethanol [C_2_H_5_OH] were procured from Merck Pvt. Ltd. India. Sodium arsenite [NaAsO_2_] and sodium arsenate [Na_3_AsO_4_] was purchased from Loba Chemie. In all experimental process, double-distilled water (DDI) was used.

### Preparation of adsorbent

#### Preparation of polystyrene (PS) sphere

A typical procedure has been followed for the preparation of PS sphere as reported in the literature^68^. According to this, 15 g of styrene monomer was added to 210 ml of DDI water and subjected to stirring at 353 K under argon atmosphere. After 10 minutes duration, 0.345 g of initiator (KPS) was added into the solution, and the reaction was continued for 24 hours followed by automatic cooling to room temperature (300 ± 3 K) to obtain PS colloid.

#### Preparation of sulfonated polystyrene (SPS) powder

The earlier synthesized monodisperse PS nanospheres were modified using concentrated sulfuric acid to allow the easy adsorption of aniline monomer on its surface^69^. In this procedure, 100 ml of earlier prepared PS colloid was subjected to sulfonation by dropwise addition of concentrated H_2_SO_4_ at 1:1 (v/v) ratio. Subsequently, it was placed in an oil bath maintained at 313 K with a continuous stirring condition for 4 hours. The product (SPS) obtained in this manner was diluted, filtered, and washed with DDI water and dried at 333 K in a vacuum oven for 12 hours.

#### Preparation of SPS@PANI sphere and PANI hollow microsphere (PNHM)

The fabrication of hollow polyaniline microsphere was carried out by dispersing 2.34 g of SPS powder directly in 230 ml DDI water under the vigorous stirring condition for 1 hour. Subsequently, 10 ml aqueous solution of aniline monomer (5 wt.% with respect to SPS) doped with 1 M HCl was added into the previous mixture at 300 ± 3 K under the stirring condition for 7–8 hours. Following this, an aqueous solution of APS (10 ml, equimolar ratio of APS to aniline) was added to it and then placed in an ice bath for further 12 hours. The resultant product was washed rapidly with DDI water and methanol followed by vacuum-dried at 323 K for 24 hours to form SPS@PANI exhibiting core-morphology. Finally, hollow polyaniline microsphere (PNHM) was fabricated by dissolving the SPS core in THF.

#### Preparation of PNHM/Fe_3_O_4_ microsphere

In a typical fabrication procedure, the desired amount of previously prepared PNHM was dispersed in ethanol followed by adding aqueous solutions of FeCl_3_.6H_2_O (7 × 10^−4^ M) and FeSO_4_.7H_2_O (3.5 × 10^−4^ M) and kept under ultrasonication for 1 hour. Thereafter, the solution was maintained at ~12 pH by dropwise addition of ammonia solution. Finally, PNHM/Fe_3_O_4_ was filtered and washed with DDI water to achieve neutral pH by removing excess ammonia and vacuum-dried at 333 K. For comparison, several PNHM/Fe_3_O_4_ composites consisting of 90/10, 80/20, 70/30, 60/40 weight percentages of PNHM/Fe_3_O_4_ (wt./wt.%) were fabricated following identical experimental procedure and designated as PNHM/Fe_3_O_4_-10, PNHM/Fe_3_O_4_-20, PNHM/Fe_3_O_4_-30 and PNHM/Fe_3_O_4_-40 respectively.

### Standard solution preparation

Standard As(III) and As(V) solution of 1000 mg L^−1^ concentration was prepared by dissolving the desired amount of NaAsO_2_ and Na_3_AsO_4_ in DDI water, respectively. The working solutions of different concentrations were freshly prepared from the stock solution to carry out batch adsorption studies.

### Adsorption experiments

Batch adsorption studies for the adsorption of As(III) and As(V) on PNHM/Fe_3_O_4_-40 were carried out to evaluate data related to equilibrium, kinetics and isotherm parameters at room temperature (300 ± 3 K) at neutral pH (~7). For this purpose, 100 ml of the arsenic solution of desired concentrations were initially taken in 250 ml capacity of polyethylene bottles (Tarson Co. Ltd., India) followed by subsequent addition of requisite amount of adsorbent dosages. After that, the test bottles were kept into BOD incubator shaker for shaking it for different time intervals at 180 ± 10 r.p.m. for all the experimental studies. The solution was filtered using 0.22 µm filter paper, and the filtrate was analyzed to calculate percentages removal of arsenic, if any, as follows^48^:

8 $$ \% \,Removal=\frac{({C}_{0}-{C}_{t})}{{C}_{0}}\times 100$$

where, *C*_0_ and *C*_*t*_ refers initial concentration (*t* = 0) and concentration at any given time (*t* = *t*), respectively. The amount of arsenic adsorbed on PNHM/Fe_3_O_4_-40 (*q*_*t*_, mg g^−1^) has been calculated according to the following equation:

9 $${q}_{t}=\frac{({C}_{0}-{C}_{t})V}{m}$$

where *m* and *V* correspond to the mass (g) of the adsorbent and the volume of arsenic solutions (L) used in each experiment. The amount of As(III) and As(V) adsorbed on PNHM/Fe_3_O_4_-40 under equilibrium *(q*_*e*_, mg g^−1^) was calculated using the following relationship:

10 $${q}_{e}=\frac{({C}_{0}-{C}_{e})V}{m}$$

where *C*_*e*_ indicates the arsenic concentration at equilibrium (mg L^−1^). The effect of variation of PNHM/Fe_3_O_4_-40 dose (0.1-6 g L^−1^ for As(III) and 0.1–2 g L^−1^ for As(V)) on the removal of arsenic was conducted at a fixed initial arsenic concentration (1000 µg L^−1^) and contact time (240 min). The kinetic study (5–420 min) was performed at fixed initial arsenic concentration and the adsorbent dose of 1000 µg L^−1^ and 1 g L^−1^, respectively. Further, the isotherm study was conducted at varying the initial concentration of arsenic (100–1000000 µg L^−1^) while keeping the fixed adsorbent dose (1 g L^−1^). The kinetics and isotherms modeling was conducted by non-linear least square method, as the various model equation is representing by non-linear relationship and linearization of those equations which is eventually associate with bias^70^. The effect on arsenic removed was also studied by carrying out experiments at different initial solution pH (3–11) at a fixed dose (1 g L^−1^) of the adsorbent. At the same time, all the experiments were duplicated to eliminate the experimental error and were taken the mean value as a final result.

### Desorption experiments

The adsorbent left after As(III) adsorption in each experiment was washed several times with DDI water. The spent adsorbent recovered in this manner was treated with 0.1 M and 0.5 M NaOH solution at room temperature (300 ± 3 K) and subjected to stirring (180 ± 10 r.p.m.) for 24 hours. Subsequently, the material was filtrated and washed with DDI water several times, and vacuum dried at 333 K for further use as an adsorbent. This desorption-adsorption study was performed four times to affirm the extent of the reusability of the material.

### Characterization technique

The morphology of the PNHM, Fe_3_O_4_, and PNHM/Fe_3_O_4_ composites was evaluated using high-resolution FE-SEM on Carl Zeiss Supra 40 instruments at an accelerating voltage of 20 kV. TEM of the samples was analyzed by using Phillips CM 200 (Netherland), with an acceleration voltage of 200 kV. FTIR and BET surface area of the materials was analyzed by using Perkin Elmer and Quantachrome Autosorb IQ instruments, respectively. XRD pattern was used to determine the crystalline phase of the material and was recorded with D8 Advance diffract meter, Bruker, Germany, with Cu K_α_ radiation (λ = 0.154 nm) with a scanning rate of 2θ = 3^0^ per min. Magnetic properties of the materials were measured at room temperature (300 ± 3 K) through physical property measurement system (PPMS) by using mini cryogen-free magnet system. XPS analysis of the samples was performed by using the PHI 5000 Versa Probe II (ULVAC–PHI, Japan) system.

Following Instruments are used during the experiment: Atomic absorption spectroscopy (AAS) [Thermo Fisher (iCE3300 AA Spectro), USA] was used to detect the arsenic and iron concentration of the samples. Ion chromatography (IC) (883 Basic IC plus, Metrohm) was used to measure the cation and anion of arsenic-contaminated groundwater sample. The pH meter (ANALAB SCIENTIFIC pH/ORP Analyzer) was used to measure the pH of the samples by using the glass electrode. Temperature and speed-controlled BOD incubator shaker were used for conducting the adsorption experiments.

## Supplementary Information

Supplementary Information.

## Data Availability

Datasets are generated during the current study.
