# Supplementary material for: Hollow Polyaniline Microsphere/Fe3O4 Nanocomposite as an Effective Adsorbent for Removal of Arsenic from Water
Source: Sci Rep. 2020 Mar 18;10:4982. doi: 10.1038/s41598-020-61763-z (PMC7080834; doi:10.1038/s41598-020-61763-z)
Supplement: Supplementary file 1 — Supplementary Information. [file 41598_2020_61763_MOESM1_ESM.pdf]

Supplementary Information

# Hollow Polyaniline Microsphere/$Fe_3O_4$ Nanocomposite as an Effective Adsorbent for Removal of Arsenic from Water

*Soumi Dutta*[1], *Kunal Manna*[2], *Suneel Kumar Srivastava*[2,3*], *Ashok Kumar Gupta*[1+], *Manoj Kumar Yadav*[4]

[1]School of Water Resources, Indian Institute of Technology Kharagpur, Kharagpur–721302, India

[2]Department of Chemistry, Indian Institute of Technology Kharagpur, Kharagpur–721302, India

[3]School of Energy Science and Engineering, Indian Institute of Technology Kharagpur, Kharagpur–721302, India

[4]School of Environmental Science and Engineering, Indian Institute of Technology Kharagpur, Kharagpur–721302, India

**For Correspondence:**

[*]S.K. Srivastava (sunil111954@yahoo.co.uk), [+]A.K. Gupta (agupta@civil.iitkgp.ac.in)

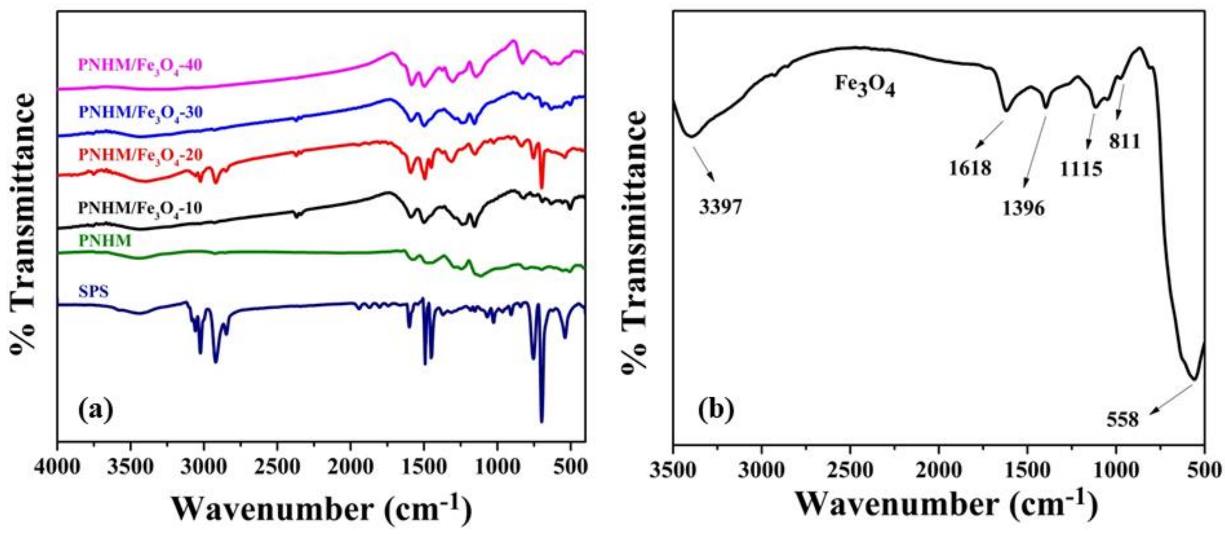


**Figure S1**. FTIR spectra of (a) SPS, PNHM and PNHM/$Fe_3O_4$ composites; (b) $Fe_3O_4$ nanoparticles.

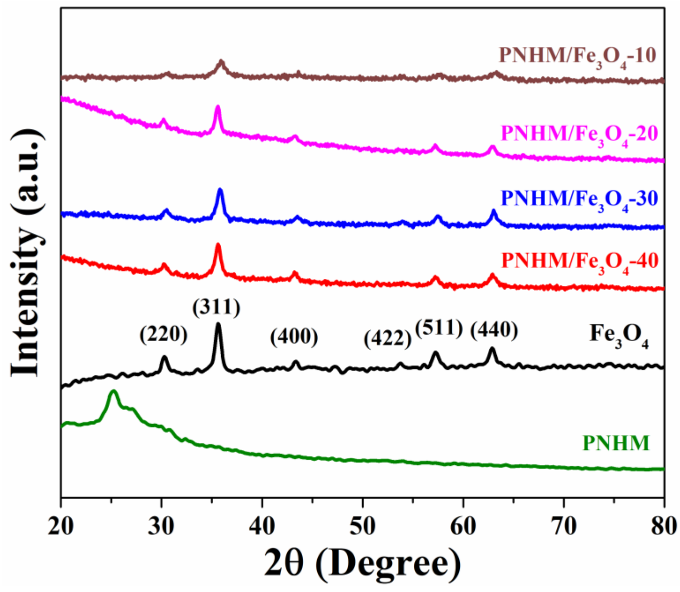


**Figure S2.** X-ray diffraction pattern of PNHM, $Fe_3O_4$ and PNHM/$Fe_3O_4$ composites.

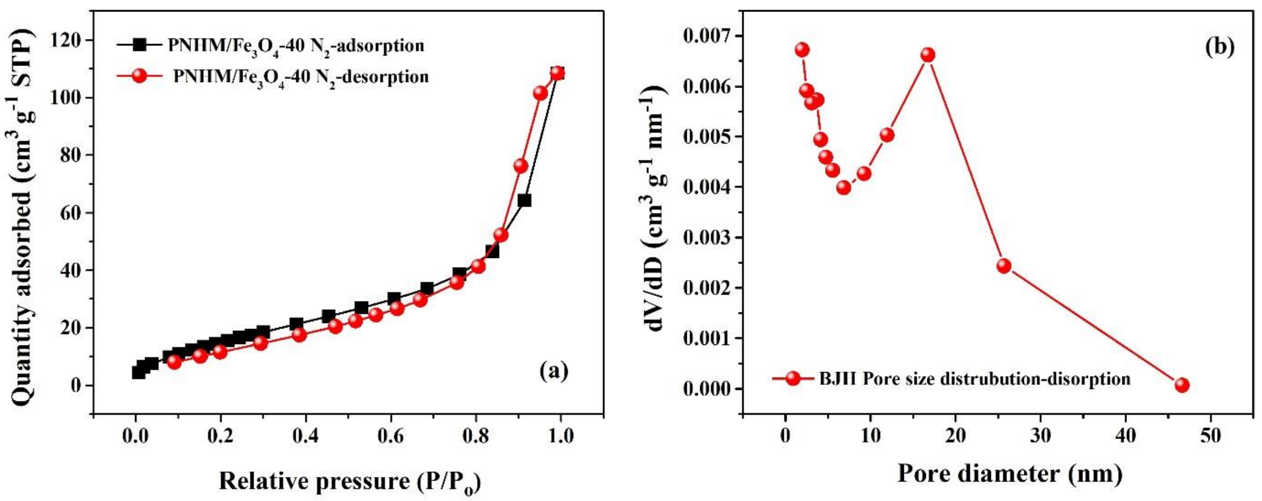


**Figure S3.** (a) $N_2$ adsorption–desorption isotherm measured for PNHM/$Fe_3O_4$–40; (b) BJH pore size distribution plot of PNHM/ $Fe_3O_4$-40.

**Table S1.** $M_s$, $M_r$, $H_c$ values of $Fe_3O_4$ and PNHM/$Fe_3O_4$-40

| Materials | Saturation magnetization, $M_s$ (emu $g^{-1}$) | Remanence, $M_r$ (emu $g^{-1}$) | Coercivity, $H_c$ (Oe) |
|---|---|---|---|
| $Fe_3O_4$ | ~66.733 | ~4.293 | ~31.336 |
| PNHM/$Fe_3O_4$–40 | ~24.398 | ~0.558 | ~30.837 |

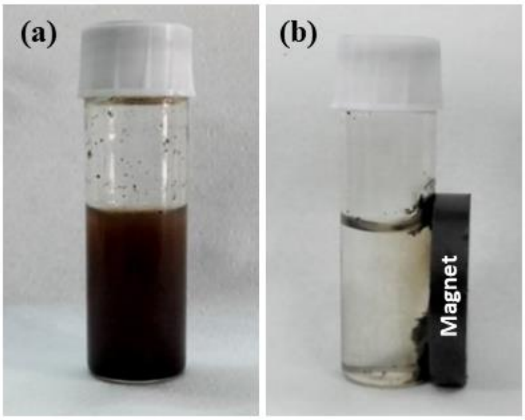


**Figure S4.** (a) Photos of PNHM/$Fe_3O_4$-40 dispersed in aqueous solution; (b) Separating by an external magnetic field.

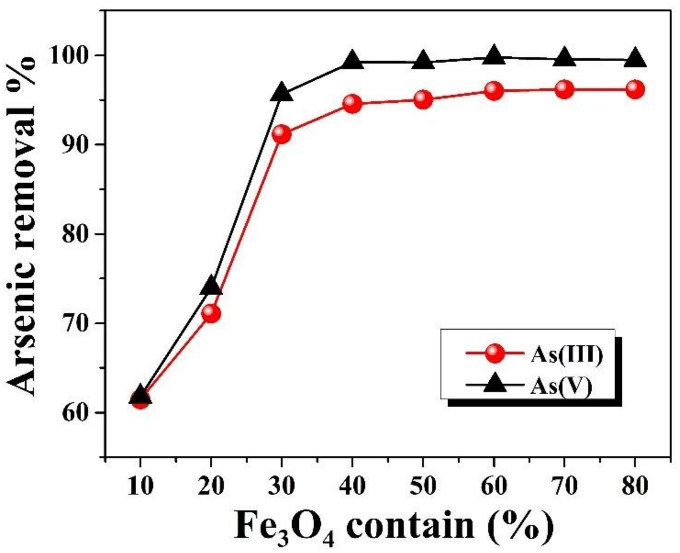


**Figure S5.** The effect of $Fe_3O_4$ content in PNHM/$Fe_3O_4$ nanocomposites on As(III) and As(V) uptake (Experimental conditions: $C_0$: 1000 µg $L^{-1}$; pH~7; adsorbent dose: 1g $L^{-1}$; contact time: 240 min; T: 300±3 K)

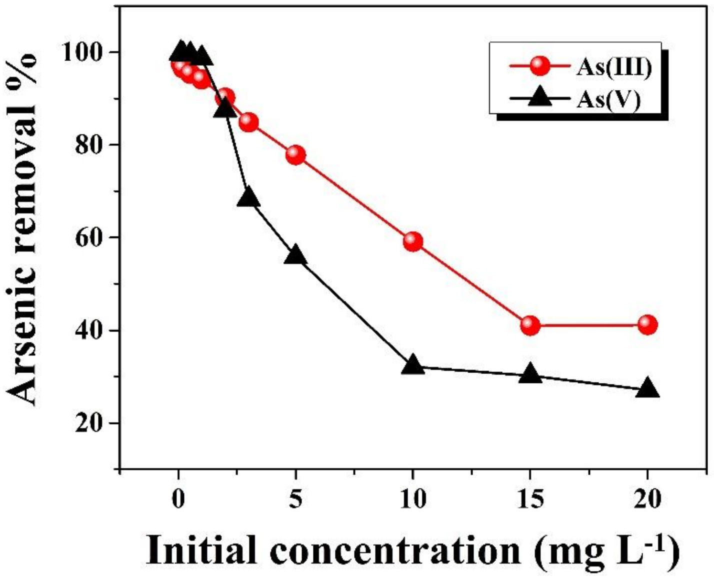


**Figure S6.** Effect of initial concentration of arsenic on removal efficiency (Experimental conditions: pH~7; adsorbent dose: 1g $L^{-1}$; Contact time: 240 min; T: 300±3 K).

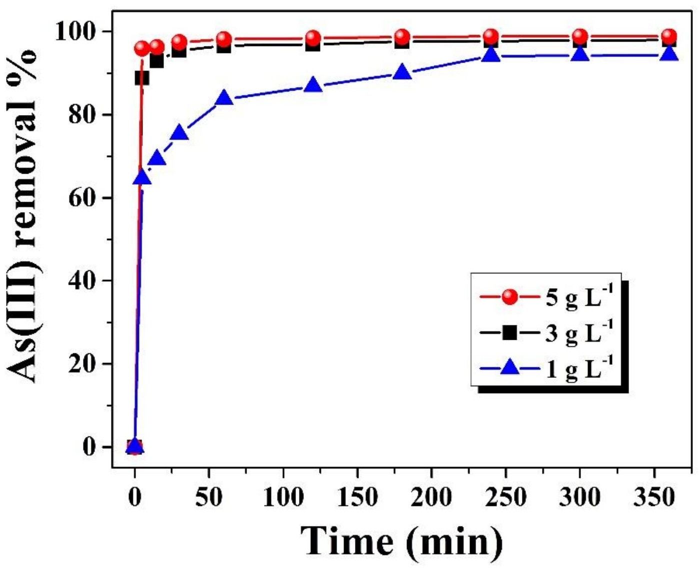


**Figure S7.** Effect of contact time on As(III) removal % at different adsorbent dose (1, 3, 5 g $L^{-1}$) with a fixed adsorbate concentration (1000 μg $L^{-1}$).

**SI–1: Adsorption kinetics.** The pseudo first-order kinetics model also referred as Lagergren first-order model, as following form[1,2]:

$$q_t = q_e(1 - e^{-k_1 t}) \tag{1}$$

where $q_e$ and $q_t$ indicates the amount arsenic uptake per unit weight (mg $g^{-1}$) of PNHM/$Fe_3O_4$-40 at equilibrium and any time $t$, respectively, and $k_1$ represents the rate constant ($min^{-1}$) of pseudo first-order model. Experimental data's are also fitted in the pseudo second-order kinetic model using the rate equation described by Ho and McKay[3] as follows:

$$q_t = \frac{(k_2\, q_e^2\, t)}{(1+k_2 q_e\, t)} \tag{2}$$

where $k_2$ is the pseudo second-order rate constant. The residual root means square error (*RMSE*) was used to measure the goodness-of-fit and was calculated by the following equation:

$$RMSE = \sqrt{\frac{\sum_{i=1}^{m}(Q_i - q_i)^2}{m}} \tag{3}$$

Where, $Q_i$ and $q_i$ indicates the observed data from the batch experiment and estimate data from kinetics and isotherm models, respectively. *m* is the number of observations taken in the experiment. It is observed that the smaller *RMSE* value indicates the better curve-fitting.

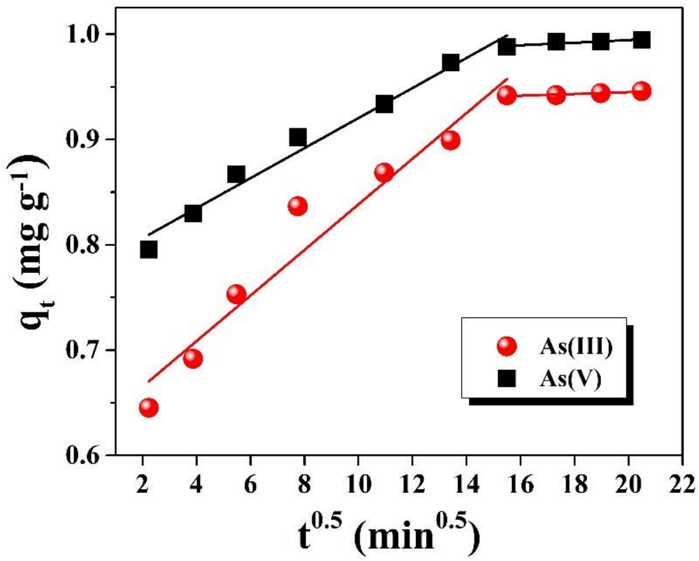


**Figure S8.** Kinetics data fitted to The Weber–Morris intra-particle diffusion plot for As(III) and As(V) adsorption on PNHM/$Fe_3O_4$-40 (Experimental conditions: adsorbent dose: 1g $L^{-1}$; $C_0$: 1000 µg $L^{-1}$; pH~7; T: 300 ± 3 K).

**Table S2.** Kinetic parameters for As(III) and As(V) adsorption on PNHM/$Fe_3O_4$-40

| Kinetic models | | Parameters | As(III) | As(V) |
|---|---|---|---|---|
| Pseudo first-order model | | $q_e$ (mg g$^{-1}$) | 0.877 | 0.943 |
| | | $k_1$ (min$^{-1}$) | 0.226 | 0.359 |
| | | $R^2$ | 0.919 | 0.964 |
| | | *Adj.* $R^2$ | 0.910 | 0.960 |
| | | *RMSE* | 0.083 | 0.058 |
| Pseudo second-order model | | $q_e$ (mg g$^{-1}$) | 0.916 | 0.966 |
| | | $k_2$ (g mg$^{-1}$ min$^{-1}$) | 0.363 | 0.733 |
| | | $R^2$ | 0.969 | 0.983 |
| | | *Adj.* $R^2$ | 0.965 | 0.982 |
| | | *RMSE* | 0.051 | 0.039 |
| Intra-particle diffusion model | Steep slope | $k_d$ (mg g$^{-1}$ min$^{-0.5}$) | 0.022 | 0.014 |
| | | *C* (mg g$^{-1}$) | 0.622 | 0.778 |
| | | $R^2$ | 0.950 | 0.979 |
| | | *Adj.* $R^2$ | 0.940 | 0.974 |
| | | *RMSE* | 0.027 | 0.011 |
| | Gradual slope | $k_d$ (mg g$^{-1}$ min$^{-0.5}$) | $8.2\times10^{-4}$ | 0.001 |
| | | *C* (mg g$^{-1}$) | 0.929 | 0.969 |
| | | $R^2$ | 0.918 | 0.848 |
| | | *Adj.* $R^2$ | 0.877 | 0.772 |
| | | *RMSE* | $6.5\times10^{-4}$ | 0.001 |

**SI–2: Adsorption isotherm.** Langmuir isotherm model is described below[4]:

$$q_e = \frac{Q_m\, b\, C_e}{1+b\, C_e} \qquad (4)$$

where, $b$ refers the Langmuir constant corresponding to the binding energy (L $mg^{-1}$) of the solute and $Q_m$ is the maximum adsorption capacity (mg $g^{-1}$). In addition, Freundlich isotherm model has been used to describe adsorption of arsenic taking place on a heterogeneous surface[5] of PNHM/$Fe_3O_4$-40 is not restricted to monolayer formations and can be described as below:

$$q_e = K_f\, C_e^{\frac{1}{n}} \qquad (5)$$

where, $K_f$ and $n$ are the Freundlich constant corresponding to the adsorption capacity [(mg $g^{-1}$)(L $mg^{-1}$) $^{1/n}$] and intensity of the adsorption, respectively.

**Table S3.** Isotherm parameters for As(III) and As(V) adsorption on PNHM/$Fe_3O_4$-40

| **Isotherm models** | **Parameters** | **As(III)** | **As(V)** |
|---|---|---|---|
| Langmuir isotherm | $Q_m$ (mg $g^{-1}$) | 28.265 | 83.078 |
| | $b$ (L $mg^{-1}$) | 0.015 | 0.002 |
| | $R^2$ | 0.907 | 0.987 |
| | *Adj.* $R^2$ | 0.901 | 0.986 |
| | *RMSE* | 3.047 | 2.212 |
| Freundlich isotherm | $K_f$ [(mg $g^{-1}$)(L $mg^{-1}$)$^{1/n}$] | 3.145 | 1.057 |
| | *1/n* | 0.326 | 0.576 |
| | $R^2$ | 0.984 | 0.999 |
| | *Adj.* $R^2$ | 0.983 | 0.998 |
| | *RMSE* | 1.264 | 0.730 |

**Table S4.** Comparison of maximum adsorption capacity of the PNHM/$Fe_3O_4$-40 with other adsorbents for removal of arsenic (pH~7)

| Type of adsorbent | Maximum adsorption capacity (mg $g^{-1}$) | | Ref. |
|---|---|---|---|
| | **As(III)** | **As(V)** | |
| $Fe_3O_4$ nanoparticles | 46.06 | 16.56 | 6 |
| α-$Fe_2O_3$ | 95.0 | 47.0 | 7 |
| $Fe_3O_4$@Polyaniline | 1.385 | 1.066 | 8 |
| polyaniline/polystyrene nanocomposite | 52 | 56 | 9 |
| Nano Zero-Valent Iron on Activated Carbon | 18.2 | 12.0 | 10 |
| Porous $Fe_3O_4$ | 6.77 | 7.23 | 11 |
| Commercial $Fe_3O_4$ | 0.76 | 1.35 | 11 |
| Poly(lauryl methacrylate divinylbenzene)/poly(glycidyl methacrylate)/$Fe_3O_4$ | 53.97 | - | 12 |
| $Fe_2O_3$@C | 29.40 | 17.9 | 13 |
| $Fe_3O_4$–Honeycomb briquette cinders | 1.566 | 1.288 | 14 |
| **PNHM/$Fe_3O_4$-40** | **28.27** | **83.08** | **Present study** |

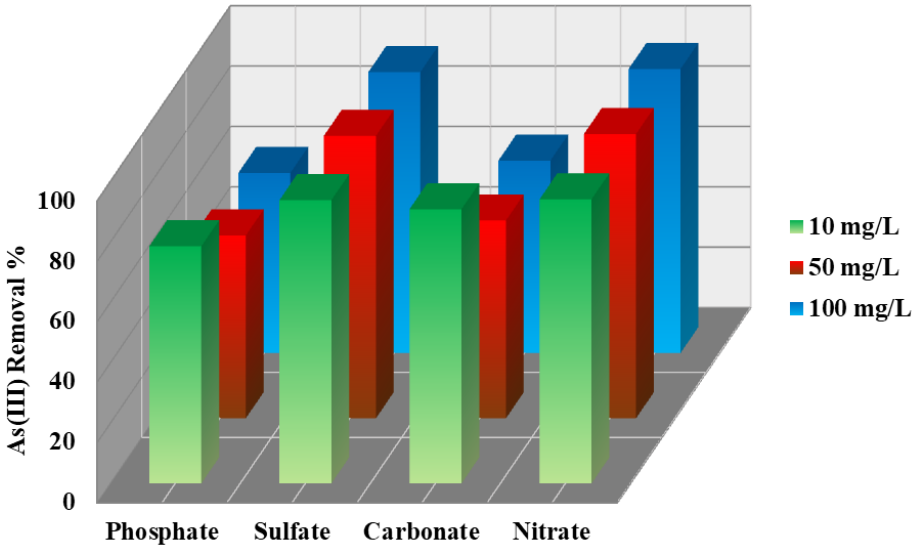


**Figure S9.** Effect of competing ions on removal of As(III) using PNHM/$Fe_3O_4$-40 (Experimental conditions: adsorbent dose: 1g $L^{-1}$; $C_0$: 1000 µg $L^{-1}$; T: 300±3 K; contact time: 240 min).

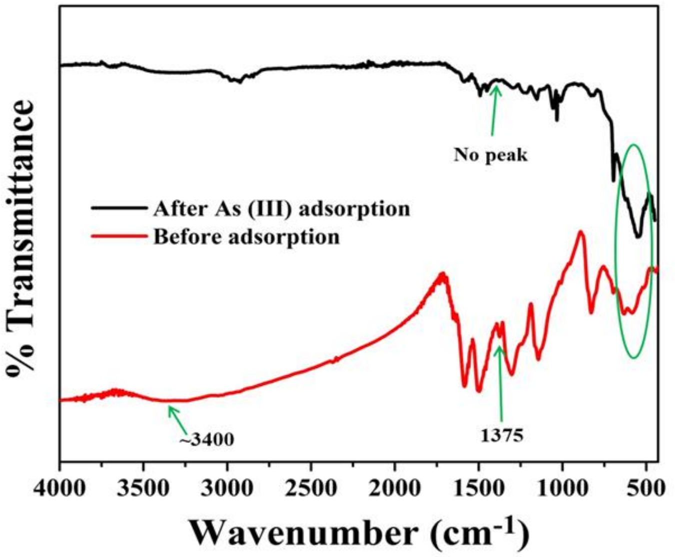


**Figure S10.** FTIR spectra of PNHM/$Fe_3O_4$-40 composite particles at before and after As(III) adsorption.

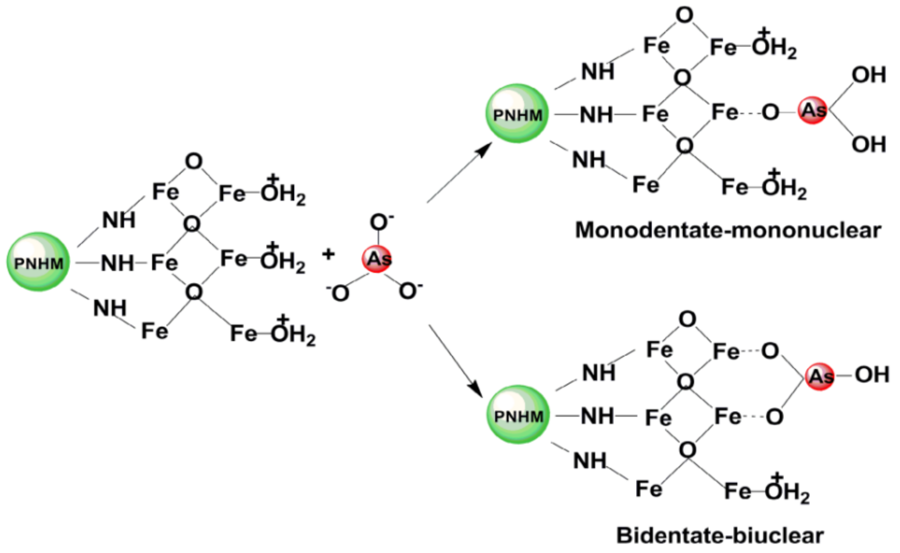


**Figure S11.** Probable mechanism of arsenic adsorption on PNHM/$Fe_3O_4$-40 at pH ~7.

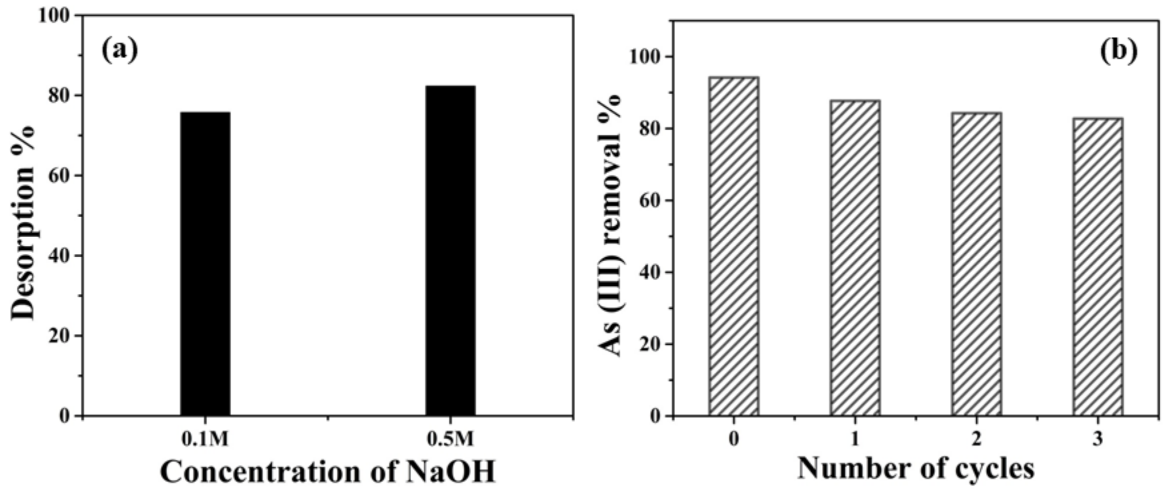


**Figure S12.** (a) Desorption of As(III) from loaded PNHM/$Fe_3O_4$–40 with different concentration of NaOH solutions; (b) As(III) removal % using regenerated PNHM/$Fe_3O_4$-40 upto three adsorption/desorption cycles (Experimental conditions: $C_0$: 1000 μg $L^{-1}$, dose 1g $L^{-1}$, T: 300±3 K , pH~7).

**Table S5.** Physicochemical parameters of naturally arsenic contaminated groundwater sample

| Parameters | Quantity |
|---|---|
| Total arsenic (μg $L^{-1}$) | 152.54 |
| Conductivity (μs $cm^{-1}$) | 2402 |
| Total dissolved solid (mg $L^{-1}$) | 1404 |
| Salinity (PSU) | 1.09 |
| pH | 7.13 |
| Turbidity (NTU) | 59.27 |
| Sodium (mg $L^{-1}$) | 239.348 |
| Potassium (mg $L^{-1}$) | 273.596 |
| Calcium (mg $L^{-1}$) | 65.920 |
| Magnesium (mg $L^{-1}$) | 0.576 |
| Chloride (mg $L^{-1}$) | 377.742 |
| Phosphate (mg $L^{-1}$) | 1.153 |
| Total alkalinity (mg $L^{-1}$) | 480 |
| Total suspended solid (mg $L^{-1}$) | 41 |

## REFERENCES


1. Ho, Y. S. & McKay, G. A Comparison of Chemisorption Kinetic Models Applied to Pollutant Removal on Various Sorbents. *Process Safety and Environmental Protection* **76**, 332–340 (1998).
2. Qiu, H. *et al.* Critical review in adsorption kinetic models. *Journal of Zhejiang University-Science A* **10**, 716–724 (2009).
3. Ho, Y. S. & McKay, G. Sorption of dye from aqueous solution by peat. *Chemical Engineering Journal* **70**, 115–124 (1998).
4. Bhaumik, M., Noubactep, C., Gupta, V. K., McCrindle, R. I. & Maity, A. Polyaniline/$Fe^0$ composite nanofibers: An excellent adsorbent for the removal of arsenic from aqueous solutions. *Chemical Engineering Journal* **271**, 135–146 (2015).
5. Srivastava, S. K., Senapati, S., Singh, S. B. & Raul, P. K. Magnetic Ni/PPy nanocomposite as effective reusable adsorbent for removal of arsenite and fluoride from contaminated water. *RSC Advances* **6**, 113424–113431 (2016).
6. Feng, L., Cao, M., Ma, X., Zhu, Y. & Hu, C. Superparamagnetic high-surface-area $Fe_3O_4$ nanoparticles as adsorbents for arsenic removal. *Journal of Hazardous Materials* **217–218**, 439–446 (2012).
7. Tang, W., Li, Q., Gao, S. & Shang, J. K. Arsenic(III,V) removal from aqueous solution by ultrafine α-$Fe_2O_3$ nanoparticles synthesized from solvent thermal method. *Journal of Hazardous Materials* **192**, 131–138 (2011).
8. Zhou, Q., Wang, J., Liao, X., Xiao, J. & Fan, H. Removal of As(III) and As(V) from water using magnetic core-shell nanomaterial $Fe_3O_4$@polyaniline. *International Journal of Green Technology* **1**, 54-64 (2015).
9. Davodi, B. & Jahangiri, M. Determination of optimum conditions for removal of As(III) and As(V) by polyaniline/polystyrene nanocomposite. *Synthetic Metals* **194**, 97–101 (2014).
10. Zhu, H., Jia, Y., Wu, X. & Wang, H. Removal of arsenic from water by supported nano zero-valent iron on activated carbon. *Journal of Hazardous Materials* **172**, 1591–1596 (2009).
11. Wang, T. *et al.* Controllable Synthesis of Hierarchical Porous $Fe_3O_4$ Particles Mediated by Poly(diallyldimethylammonium chloride) and Their Application in Arsenic Removal. *ACS Applied Materials & Interfaces* **5**, 12449–12459 (2013).
12. Shabnam, R. *et al.* Novel Magnetically Doped Epoxide Functional Cross-linked Hydrophobic Poly(lauryl methacrylate) Composite Polymer Particles for Removal of As(III) from Aqueous Solution. *Industrial & Engineering Chemistry Research* **56**, 7747–7756 (2017).
13. Wu, Z., Li, W., Webley, P. A. & Zhao, D. General and controllable synthesis of novel mesoporous magnetic iron oxide@carbon encapsulates for efficient arsenic removal. *Advanced Materials* **24**, 485–491 (2012).
14. Baig, S. A. *et al.* Arsenic removal from aqueous solutions using $Fe_3O_4$-HBC composite: effect of calcination on adsorbents performance. *PloS one* **9**, e100704 (2014).
